# Approach to Neonates and Young Infants with Fever without a Source Who Are at Risk for Severe Bacterial Infection

**DOI:** 10.1155/2018/4869329

**Published:** 2018-11-26

**Authors:** Susanna Esposito, Victoria Elisa Rinaldi, Alberto Argentiero, Edoardo Farinelli, Marta Cofini, Renato D'Alonzo, Antonella Mencacci, Nicola Principi

**Affiliations:** ^1^Pediatric Clinic, Department of Surgical and Biomedical Sciences, Università degli Studi di Perugia, Perugia, Italy; ^2^Microbiology Unit, Department of Medicine, Università degli Studi di Perugia, Perugia, Italy; ^3^Pediatrics, Università degli Studi di Milano, Milan, Italy

## Abstract

**Introduction:**

Among neonates and infants <3 months of age with fever without a source (FWS), 5% to 15% of cases are patients with fever caused by a serious bacterial infection (SBI). To favour the differentiation between low- and high-risk infants, several algorithms based on analytical and clinical parameters have been developed. The aim of this review is to describe the management of young infants with FWS and to discuss the impact of recent knowledge regarding FWS management on clinical practice.

**Materials and Methods:**

PubMed was used to search for all of the studies published over the last 35 years using the keywords: “fever without source” or “fever of unknown origin” or “meningitis” or “sepsis” or “urinary tract infection” and “neonate” or “newborn” or “infant <90 days of life” or “infant <3 months”.

**Results and Discussion:**

The selection of neonates and young infants who are <3 months old with FWS who are at risk for SBI remains a problem without a definitive solution. The old Rochester criteria remain effective for identifying young infants between 29 and 60 days old who do not have severe bacterial infections (SBIs). However, the addition of laboratory tests such as C-reactive protein (CRP) and procalcitonin (PCT) can significantly improve the identification of children with SBI. The approach in evaluating neonates is significantly more complicated, as their risk of SBIs, including bacteremia and meningitis, remains relevant and none of the suggested approaches can reduce the risk of dramatic mistakes. In both groups, the best antibiotic must be carefully selected considering the clinical findings, the laboratory data, the changing epidemiology, and increasing antibiotic resistance of the most common infectious bacteria.

## 1. Introduction

Fever in neonates and infants <3 months of age is defined as a rectally obtained temperature ≥ 38°C [1–3]. Fever is one of the most common reasons for emergency department and outpatient clinic visits by these patients; many of which have no diagnostically reliable signs and symptoms and receive a diagnosis of fever without a source (FWS) after initial clinical evaluation. These infants have been divided into two groups for many years. The first group includes patients suffering from mild, clinically irrelevant viral infections and the second group, accounting for 5% to 15% of cases, includes patients with fever caused by a serious bacterial infection (SBI), i.e., invasive diseases (bacteremia/sepsis, meningitis) or severe, exceptionally invasive bacterial infections (pneumonia, urinary tract infection (UTI), and soft tissue and bone infections) [4]. Although difficult, the differentiation of neonates and young infants at risk of SBI from those without significant clinical problems is considered crucial. Early identification and treatment of patients with SBI is deemed essential to assuring favourable disease outcomes. Moreover, the selection of patients with low risks of SBI could permit the avoidance of unnecessary antibiotic treatments, hospitalization, and invasive laboratory tests.

To favour the differentiation between low- and high-risk infants, several algorithms based on analytical and clinical parameters have been developed [5–8]. However, while they were quite similar in some steps, these algorithms differed in their use of some diagnostic procedures, triggering a lively debate among the authors. Consequently, they were not systematically used in clinical practice [3, 9, 10] and were frequently substituted by homemade guidelines in many children's hospitals, which occurred in the USA [11]. The debate on the best approach to infants with FWS has been further stimulated in the past 15 years by evidence of progressively changing epidemiology, aetiology, and characteristics of SBIs [12–14]. Moreover, with time, new biomarkers have become available and their inclusion in the algorithms was thought to potentially significantly improve their diagnostic efficacy [15–17]. The main aim of this study is to describe the historical approach for young infants with FWS and to discuss the impact of recent knowledge regarding this topic on clinical practice.

## 2. Materials and Methods

PubMed was used to search for all of the studies published over the last 35 years using the keywords: “fever without source” or “fever of unknown origin” or “meningitis” or “sepsis” or “urinary tract infection” and “neonate” or “newborn” or “infant <90 days of life” or “infant <3 months”. More than 1400 articles were found, but only those published in English or providing evidence-based data were included in the evaluation.

## 3. Traditional Management of Young Infants with Fever without a Source (FWS)

In the ‘80s, the evaluation of young infants with FWS was mainly devoted to excluding subjects with SBI to reduce hospitalization rates, laboratory tests, and antibiotic consumption. The first study regarding this approach was published by Dagan et al., and the results led to the formulation of the so-called Rochester criteria [7]. The risk of SBI was deemed low in all infants who otherwise appeared well (i.e., absence of tachypnea, dyspnea, tachycardia, bradycardia, lethargy, and decreased activity/appetite), had no evidence of ear, soft tissue, or skeletal infections, and had white blood cell (WBC) counts between 5000 and 15,000/mm^3^, bands less than 1500/mm^3^, and ≤10 WBC per high-power field (HPF). Moreover, in cases with diarrhoea, SBI could be excluded if ≤5 WBC/HPF could be observed in the stool. This conclusion was drawn from evidence that, among the 144 infants that these authors classified as being low-risk because they met all these criteria, only one (0.7%) had an SBI compared to 22 (25%) of 89 infants that were included in the high-risk group (*p* < 0.0001). The test was calculated to have a sensitivity (SE) of 0.924 (95% confidence interval (CI), 0.84–0.97), a specificity (SP) of 0.499 (95% CI, 0.47–0.53), a positive predictive value (PPV) of 0.123 (95% CI, 0.10–0.16), and a negative predictive value (NPV) of 0.989 (95% CI, 0.97–1.00). Beginning with these findings, the authors of the Rochester protocol suggested that children included in the low-risk group could remain at home without antibiotics, although a follow-up was required [7]. In contrast, high-risk children should be hospitalized and receive empiric antibiotics together with further analysis and controls [7].

The accuracy of the Rochester criteria for the identification of low-risk young infants was tested in several studies [18–20], which yielded results similar to those reported by Baskin et al. [6]. In detail, the NPV and PPV values reportedly varied from 93.8% to 98.9% and 12.3% to 35.1%, respectively, confirming that only a marginal number of children with SBI could be included in the low-risk group using these criteria, although the identification of those with SBIs was suboptimal and a relevant number of patients without true clinical problems were hospitalized. However, despite these favourable results, some experts considered the Rochester protocol to be inadequate for selection. These criteria could not be applied to premature infants or to children with previously diagnosed medical conditions because these subjects were not included in the study by Baskin et al. [6]. Moreover, neonates were not distinguished from infants, although the risk of SBIs was repeatedly found higher in the first days of life than later in life [20–22]. Finally, Rochester criteria did not consider the risk that neonates and young infants with bacterial meningitis and pneumonia could be included in the low-risk group because these diseases are difficult to diagnose in the first weeks of life if only clinical findings and blood tests are used as suggested by Rochester criteria [23, 24].

To overcome these problems, several alternative methods for the identification of febrile children at low or high risk of SBI were developed. The Philadelphia [5] and Boston [6] protocols were devoted to infants aged 29–89 days old, whereas Milwaukee criteria [25] were for children 28–56 days old. Moreover, experts with expertise in pediatrics and infectious diseases or emergency medicine prepared a guideline to approach FWS in neonates [26]. In the Philadelphia, Boston, and Milwaukee statements, clinical criteria, blood tests, and cut-off levels for stratification were quite similar to those included in the Rochester protocol. The urine evaluation was not substantially different, although some additional details for excluding UTIs were included, specifically in the Milwaukee protocol. However, the most relevant difference was the inclusion of cerebrospinal fluid (CSF) testing and, when obtained, the chest radiograph. To be considered low-risk for SBI, an infant had to have a CSF WBC/mm^3^ of <8–10 and a normal chest radiograph. For neonates, Baraff et al. included a complete evaluation for sepsis, considering blood, urine, and CSF bacterial cultures, which had to all be negative for the low-risk definition [26].

Despite these modifications, these protocols did not significantly increase the ability of Rochester criteria to identify febrile children with and without SBI. Hui et al. evaluated all the literature regarding the diagnostic accuracy of different SBI screenings in febrile infants aged 3 months or younger published between 1950 and September 2010. These authors concluded that all previously cited protocols were similar for correctly identifying infants without SBI [27]. The SE ranged from 84.4% to 100%, and the NPV ranged from 93.7% to 100%. However, the SP was low, ranging from 26.6% to 69.9%. These findings were confirmed when bacteremia and meningitis were considered separately. Comparison of bacteremia SE and NPV values among the Rochester [7, 18–20, 28], Boston [29], and Philadelphia [5, 19, 30–32] criteria revealed SE values ranging from 75.0% to 100% and NPV values ranging from 97.1% and 100%. Regarding meningitis, the Philadelphia criteria [30, 31] were found to have excellent SE and NPV (100%) values but very low values for SP, varying from 24.2% to 50.7%. Finally, these protocols did not simplify the management of infants. For high-risk subjects, hospitalization and empiric antibiotic administration remained the choice with all the protocols. Low-risk infants tested with Philadelphia criteria were managed according to Rochester criteria [29]. In contrast, management of these infants became more complicated when the Boston [6] and Milwaukee [25] criteria were used because with both of these criteria, infants were sent home with prescriptions for empiric therapy (Boston) or 50 mg/kg ceftriaxone i.m. (Milwaukee).

Considering the results of the protocols derived from the Rochester protocol, the use of obtaining cultures from infants between 1 and 3 months of age to improve the efficacy of Rochester criteria was strongly criticized by some experts [33, 34]. Protocols including cultures were considered too complicated and invasive and not utilizable by primary care pediatricians and some hospital emergency departments. Particularly, the use of universal CSF testing was debated. The risk that some children aged 29–59 days with bacterial meningitis could be included in the low-risk group and have delayed diagnoses when CSF testing was lacking was considered practically inexistent. Moreover, it was evidenced that lumbar puncture could lead to higher risks of procedural complications and hospitalization of otherwise low-risk infants [35–37]. However, the low incidence of bacterial meningitis in this age group and the accuracy of Rochester criteria for the identification of children at low risk for SBIs, including bacterial meningitis, were once again confirmed by recent evaluations. Chua et al. conducted a difference-in-difference analysis to compare 7 hospitals with clinical practice guidelines recommending CSF testing for febrile infants aged 29–56 days with 25 hospitals without such guidelines [11]. In these infants, the occurrences of bacterial meningitis diagnosis, mechanical ventilation, central venous catheter placement, extracorporeal membrane oxygenation, and in-hospital mortality were uncommon and similarly distributed between the groups. This was considered evidence that even when not using CSF testing, providers could identify febrile infants at high risk for bacterial meningitis. Similar results were reported by Scarfone et al. [38]. These authors performed a retrospective cohort study in which 1188 febrile infants aged 29–56 days were enrolled. They found that only one child (0.08%) had bacterial meningitis. However, he did not meet the Rochester criteria for the low-risk SBI classification and was hospitalized and treated as needed. Unlike children aged 29–59 days, CSF testing to exclude meningitis remained mandatory for neonates, as initially proposed by Baraff et al. [26] and later suggested by the American College of Emergency Physicians [39].

Table 1 summarizes the historical criteria used in young infants with FWS to identify those at risk of SBI.

## 4. Adjunctive Diagnostic Tests to Improve the Screening of Young Infants with Fever without a Source (FWS)

To improve the prediction of SBI in children < 90 days old, several diagnostic tests used alone or in combination with those previously used were suggested. Evaluation of WBC counts with criteria different from those used in traditional screening methods was found unsatisfactory. Gomez et al. reported that among 3034 infants 22–90 days old that appeared well but presented with FWS, those with leukopenia (WBC < 5000 cells/mm^3^) exhibited a lower prevalence of UTIs (8.1% vs. 14.7%; odds ratio (OR), 0.51; 95% CI, 0.29–0.88) but a similar prevalence of invasive bacterial diseases (2.5% vs. 2.0%; OR, 1.20; 95% CI, 0.44–3.44) compared with those without leukopenia [40]. Leukopenia was only associated with a high prevalence of bacteremia and meningitis in infants not appearing well, which were included in the high-risk group by definition (17.8% vs. 6.9%; OR, 2.90; 95% CI, 1.06–7.78).

The most studied markers were C-reactive protein (CRP) and procalcitonin (PCT). In most of the studies, both markers had higher SE and SP than WBC count in the identification of SBI. Nosrati et al. reported that WBC counts, absolute neutrophil counts (ANC), and CRP levels were independent laboratory predictors of SBI in a group of 48 infants aged < 90 days with SBI [41]. However, the accuracy of CRP was significantly higher, as evidenced by the area under the receiver operating characteristic (ROC) curve (AUC).

PCT was frequently found to be even more sensitive than CRP [42–50], although in some studies, the superiority of PCT was evidenced only for the identification of invasive diseases and not for all SBIs [51, 52]. In a recent investigation in which the diagnostic characteristics of the PCT assay, CRP concentrations, WBC counts, and ANC counts for the detection of SBIs were evaluated, it was shown that although the area under the ROC curves for CRP and PCT were similar (AUC 0.81, 95% CI: 0.83–0.99 vs. AUC 0.80, 95% CI: 0.75–0.85; *p* = 0.70), PCT could more accurately detect bacteremia and bacterial meningitis (AUC 0.91, 95% CI: 0.83–0.99 vs. AUC 0.77, 95% CI: 0.65–0.89, *p* = 0.002) [52]. Moreover, interestingly, no difference was found in neonates compared to older infants. In this study, a cut-off PCT value of 0.3 ng/mL was associated with a negative likelihood ratio of 0.3 (95% CI, 0.2–0.5) for identifying SBI and 0.1 (95% CI, 0.03–0.4) for identifying bacteremia and meningitis. However, a meta-analysis of studies assessing the relevance of the 0.3 ng/mL PCT cut-off value for the identification of low- and high-risk children with FWS concluded that measuring serum PCT concentrations alone was inferior to the Rochester prediction rules even though it could differentiate some subjects [53].

Considering these limitations, the combination of more biomarkers should have increased the ability of protocols to stratify infants with FWS. Bressan et al. used a laboratory score originally derived and validated by Lacour et al. [54] that combines CRP, PCT, and urine dipstick results [55]. Two points were attributed to PCT ≥ 0.5 ng/mL or CRP ≥ 40 mg/L, 4 points to PCT ≥ 2 ng/mL or CRP ≥ 100 mg/L, and 1 point to a positive urine dipstick (i.e., positive leukocyte esterase and/or positive nitrate). A score ≥ 3 had positive and negative likelihood ratios for SBI prediction of 10.2 (95% CI, 9.5–10.9) and 0.5 (95% CI, 0.5–0.5), respectively. When only bacteremia and meningitis were considered, these values dropped to 4.3 (95% CI, 4.0–4.6) and 0.4 (95% CI, 0.3–0.5), respectively, because 30% of children with these diseases were not identified, suggesting that the laboratory score was not completely satisfactory for SBI prediction. However, when CRP and PCT were evaluated together with patient and clinical characteristics in the so-called step-by-step approach, better results were obtained [56]. With the step-by-step approach, children were divided according to clinical appearance and age and if they appeared well and were older than 21 days, they underwent laboratory tests for CRP, PCT, and ANC. Comparison of the impacts of laboratory tests, Rochester criteria, and the step-by-step approach revealed that well-appearing infants < 21 days of age with CRP < 20 mg/L, PCT < 0.5 ng/mL, and ANC < 10,000/mm^3^ had significantly lower risks of SBI. The SE and NPV of the step-by-step approach were 92% and 99.3%, respectively, compared to 81.6% and 98.3% of the Rochester criteria and 59.8% and 98.1% of the laboratory score.

The addition of CRP and PCT to old screening protocols did not significantly reduce the risk that well-appearing younger infants are considered at-risk and receive unneeded therapy. Rochester criteria and other older screening tests have very good NPV values that are only marginally increased by the addition of CRP and PCT. On the contrary, as evidenced by the study by Gomez et al. [15], the addition of these biomarkers seems useful in infants 29–90 days old who are categorized as at-risk with Rochester criteria. In this case, a greater number of patients with true SBI are promptly identified and can receive a more appropriate clinical approach and therapy, particularly when more than one laboratory test is used. Moreover, a greater number of infants without SBI are identified and can be sent home without the need for hospitalization. However, the advantages for neonates are marginal because CRP and PCT are influenced by several factors other than SBIs. Regardless of laboratory test results, neonates with FWS continued to be immediately hospitalized, receive complete evaluation for sepsis, and be treated with antibiotics.

Advances in the identification of SBIs in neonates and young infants might be due to some recently identified biomarkers of sepsis. Studies seem to indicate that soluble triggering receptor expressed on myeloid cells-1 (sTREM-1), interleukin- (IL-) 27, soluble urokinase plasminogen activator receptor (suPAR), neutrophil CD64, presepsin, cell-free DNA (cfDNA), and microRNA (miRNAs) can have relevant roles in this regard [57, 58]. Most studies concerning these biomarkers have been carried out to evaluate their ability to identify adults with systemic inflammatory response syndrome (SIRS). However, pediatric data are also available for some of these biomarkers. Wong et al. studied IL-27, a cytokine produced by antigen-presenting cells after exposure to microbes and inflammatory stimuli, and found that at serum cut-point values ≥ 5 ng/mL, this marker predicted SBIs in children < 10 years old, thus meeting pediatric-specific criteria for SIRS, sepsis, and septic shock with SP and PPV values > 90% and an overall performance generally better than that of PCT [58]. However, the potential role of IL-27 for the stratification of children with suspected infection was only partly confirmed by Hanna et al. [59]. These authors studied children < 15.1 years old who were admitted to the pediatric intensive care unit for SBI and found the PPV of this biomarker for bacterial infections with FWS to be modest. In contrast, bacteremia was identified in almost all cases with positive blood cultures (SP 95%; 95% CI, 92%–96%).

Presepsin is a glycoprotein that serves as a receptor for bacterial lipopolysaccharides and activates proinflammatory responses upon exposure to these agents [60]. Presepsin is considered a potential marker of sepsis in neonates because most variables that commonly affect CRP and PCT in these subjects do not affect presepsin levels [61]. This biomarker was found at higher levels in neonates with late-onset sepsis than in healthy matched subjects (median value, 1295 vs. 562 ng/L, *p* = 0.00001). The best calculated cut-off value was 885 ng/L, with an SE of 94%, an SP of 100%, a negative likelihood ratio 0.05, and an infinite positive likelihood ratio [62].

Further improvements in the identification of SBIs can potentially be obtained using a genomic approach. Because bacteria induce specific host responses that can be detected using a microarray analysis of leukocytes [63–66], analysis of this response could lead to the distinction of bacterial and viral infections. Studies carried out in older children and adults have confirmed this supposition with >95% accuracy [67–71]. Moreover, a recent study documented that despite their immature immune systems, even children < 60 days old respond with RNA biosignatures that permit the differentiation of bacterial and viral infections and discriminate the class of pathogens that cause the infection [72]. A total of 66 classifier genes capable of distinguishing infants with and without SBI were identified, with an SE of 87% (95% CI, 73–95) and an SP of 89% (95% CI, 81–83). Bacteremia was associated with 10 classifier genes with an SE of 94% (95% CI, 70–100) and SP of 95% (95% CI, 88–98).

Recently, new syndromic molecular methods have been developed for rapid etiologic diagnosis of localized or systemic infections. These multiplex real-time polymerase chain reaction tests are able to detect, directly in a single clinical specimen, the most important viral, bacterial, or fungal pathogens responsible for gastrointestinal infections [73], respiratory infections [74], sepsis [75, 76], or meningitis [77] and are characterized by a good diagnostic accuracy. However, their clinical impact and the possibility to include them in the diagnostic algorithms for infants with FWS have not been thoroughly evaluated yet. In this respect, it is noteworthy that both PCT and presepsin have been proved to predict molecular results in adult patients with sepsis [78, 79].

In addition, the Roche SeptiFast® MGRADE PCR with a modified DNA extraction protocol and software-handling tool was tested for the identification of neonatal SBI. Results were compared to blood culture, laboratory biomarkers, and clinical signs of sepsis [80]. Data highlighted that the Roche SeptiFast® MGRADE PCR using a modified DNA extraction protocol appears useful for rapid detection of neonatal sepsis in addition to conventional blood culture. However, this method has a high risk of contamination, does not permit to evaluate antibiotic resistance, and is quite expensive [80].

For all these new approaches, additional studies with larger populations are needed to define the accuracy estimates and to assess their possible use in neonates and infants < 90 days old with FWS. However, it must be remembered that most SBIs in this age group are attributable to UTIs and these new approaches that are characterized by high costs are mainly devoted to the identification of bacteremia and meningitis, although no information is available on their efficacy in the evaluation of these diseases.

## 5. Factors Modifying the Approach to Fever without a Source (FWS) in Neonates and Young Infants

### 5.1. Role of Urinary Tract Infection (UTI)

When Rochester criteria were developed, bacteremia was the most common SBI in young infants, accounting for approximately 20%–30% of all cases. Meningitis was diagnosed in 0%–14% of the cases, and UTI was observed in 30%–55% of the cases [5, 20]. A recent analysis of cultures obtained from full-term neonates and infants 1 week to 3 months of age who received care at Kaiser Permanente Northern California from 2007 to 2011 revealed that while no change in the overall SBI rate was observed, the distribution of SBIs has significantly changed in the past 20 years [12]. The prevalence of SBIs due to isolated bacteremia and meningitis was quite low, as it was limited to 6.3% and 0.2%, respectively. In contrast, UTI was more common, as it was observed in 92.2% of SBI cases (84% alone and 8.2% in combination with other infections). The increase in UTI frequency and the consequential need for the early detection of these diseases have raised the problem of the accuracy of traditional UTI test diagnostics. In the first screening tests, UTI was excluded simply based on the absence of significant pyuria, although some UTIs were known to be characterized by little or no pyuria and the threshold suggesting a true UTI was not definitively established [81]. Later, to exclude a UTI, some authors suggested including negative leukocyte esterase and negative nitrite criteria in the urinalysis. However, whether dipstick urinalysis can be an accurate predictor of UTI is debated. In a retrospective study [82] of children from 2 months to 2 years old who presented to the emergency department with fever and a positive urine culture, only 69.9% of the patients with culture-proven UTIs had a positive urinalysis. Positive leucocyte esterase and nitrite were detected in 63.5% and 20.9% of the cases, respectively, suggesting that dipstick analysis adds little to the microscopic evaluation of pyuria independent of the suggested urine WBC thresholds [4–6]. On the contrary, Tzimenatos et al. [83] have recently found that among children aged ≤ 60 days with FWS and urine culture with ≥50,000 CFUs/mL, a positive urinalysis by the presence of any leukocyte esterase, nitrite, or pyuria exhibited sensitivity of 0.94 (95% CI: 0.91–0.97) and specificity of 0.91 (95% CI: 0.90–0.91) in all groups. An advance could be assured by the incorporation of urine concentration into the interpretation of automated microscopic urinalysis that is usually performed to evaluate the possible presence of UTI.

A recent study determined which urine WBC threshold was most likely associated with documented UTIs according to urine concentration [84]. The optimal WBC cut-offs were 3 WBC/HPF in urine samples with specific gravities < 1015 and 6 WBC/HPF in urine samples with higher specific gravities. The likely positive and negative ratios were 9.9 and 0.15 for diluted urine and 10.1 and 0.17 for concentrated urine, respectively. However, it must be remembered that UTIs are correctly diagnosed only by urine culture, a number of young infants with UTIs have negative urinalysis, and only about 5% of UTIs in pediatric-aged children are associated with bacteremia and have urosepsis [85]. It is worth noting that in UTI matrix-assisted time-of-flight mass spectrometry performed directly in urine is able to identify pathogens in about 1 hour [86], suggesting that, in the near future, this technology could be included in the routine diagnostics, anticipating culture results.

### 5.2. Role of Viral Infection

The study that had led to the definition of Rochester criteria also reported that a relevant number of young infants included in both the low- and high-risk groups had viral infections [7]. Unfortunately, this information could not be included among the criteria for screening because of the lack of timely results. As later evidence, the incorporation of viral information into the risk criteria could have significantly improved the stratification capacity of the protocol. Several studies have highlighted that association between confirmed viral infections and most SBIs, including bacteremia and meningitis, is generally uncommon. Only UTI prevalence was found to not always be significantly influenced by the coexistence of viral infection. Byington et al. studied the distribution of infections due to enteroviruses, respiratory viruses, rotaviruses, and herpesviruses among 1385 infants aged < 90 days with FWS who were classified as low- or high-risk for SBIs according to Rochester criteria [87]. These authors reported that the SBI occurrence was significantly lower in infants with viral infections than in those without (4.2% vs. 12.3%; *p* < 0.0001). High-risk virus-positive patients had significantly fewer SBIs than high-risk virus-negative patients (5.5% vs. 16.7%; *p* < 0.0001). When compared with high-risk virus-negative patients, high-risk virus-positive patients were less likely to have bacteremia, UTIs, or soft tissue infections and high-risk virus-positive patients had bacteremia occurrences similar to those of low-risk patients (0.92% vs. 1.97%; *p* = 0.24) but higher occurrences of UTI (3.7% vs. 1%; *p* = 0.002).

The relationship between RSV infection and the development of SBIs was investigated by Levine et al. in a group of 1248 febrile infants ≤ 60 days old [88]. SBIs were significantly more common in RSV-negative children (12.5%; 95% CI, 10.5–14.8) than in those with RSV infection (7.0%; 95% CI, 4.1–10.9; relative risk (RR), 0.6; 95% CI, 0.3–0.9). These findings were partly confirmed when single SBIs were analysed, as RSV-positive infants had a lower rate of bacteremia than RSV-negative infants, although the differences were not significant (1.1% vs. 2.3%; risk difference, 1.2%; 95% CI, −0.4% to 2.7%). No RSV-positive infants had bacterial meningitis (0 of 251; 95% CI, 0%–1.2%). However, more than 5% of these patients were found to suffer from UTIs. The very low risk of SBIs in infants suffering from bronchiolitis and RSV infection was further evidenced by Ralston et al. [89]. These authors analysed all the studies published until December 31, 2010, in which rates of UTI, bacteremia, and meningitis in younger infants with bronchiolitis and RSV infection had been evaluated. It was found that the weighted rate of UTIs in the 11 studies analysed was 3.3% (95% CI, 1.9–5.7). Bacteremia was observed in 3 of 11 studies. No case of meningitis was reported in any of the studies. Similar results were reported when the association between influenza and SBIs was evaluated. In a study testing 809 children ≤ 60 days old, the risk of SBI was significantly higher in subjects without influenza than in those with this infection (13.3%, 95% CI: 10.9–16.1 vs. 2.5%, 95% CI: 0.5–7.2; *p* < 0.001) [90]. Bacteremia and meningitis were diagnosed in 2.2% and 0.9% of influenza-negative infants, respectively, and in no infants with influenza, with no statistically significant differences between the groups. However, the prevalence of UTI was significantly higher in influenza-negative children than in those that were influenza-positive (10.8%, 95% CI: 8.6%–13.3% vs. 2.4%, 95% CI: 0.5–6.9; *p* = 0.002).

All these findings provide evidence that when several rapid methods for detecting viruses are available, the addition of viral diagnostics to Rochester criteria could permit to better classify patients regarding their individual risk for SBI. For a complete evaluation, the herpes simplex virus and parechovirus must be studied together with traditionally tested viruses, as these viruses can be associated with very severe, life-threatening clinical manifestations. Recent data have highlighted the relevance of these agents as causes of FWS in neonates and young infants [91, 92]. Virus detection can lead to significant advantages for both infants and the health care system. The implementation of an evidence-based care process model for the care of febrile infants, including viral tests, was associated with the increased delivery of evidence-based care and the reduction of hospital stay, time exposed to antibiotics, and general costs of health services [93].

However, it cannot be forgotten that evidence of a coexisting viral infection does not exclude the presence of an SBI, although this evidence is useful for better selection. A small, but significant, number of neonates and young infants are not correctly categorized even with the addition of viral tests. Moreover, viral detection in respiratory secretions does not systematically indicate that the virus is the true cause of the actual disease. Several studies have demonstrated that some respiratory viruses are shed for several days after the disease is resolved [94, 95], which can mask the true aetiology of the disease.

### 5.3. Changing the Distribution of Infectious Bacteria

In older studies, numerous SBIs, particularly those diagnosed in neonates, were due to group B *Streptococcus* (GBS) and *Listeria monocytogenes*. However, recent studies have clearly evidenced that rates of early-onset GBS and *Listeria monocytogenes* infections have been significantly reduced. Moreover, significant modifications in the distribution of bacterial pathogens causing late-onset infections have been reported. Together with a relevant reduction of late-onset GBS infections and the almost complete disappearance of *Listeria monocytogenes* infection, epidemiological studies have clearly shown that Gram-negative rods, mainly *Escherichia coli*, were the most common causes of SBIs in children aged 7–90 days, as they were detected not only in UTIs but also in bacteremia and meningitis [96, 97]. The cause for this shift is not completely known. The reduction of SBI cases due to *Haemophilus influenzae* type b and *Streptococcus pneumoniae* in young infants might be due to the herd immunity effect resulting from the use of conjugate vaccines against these pathogens in the pediatric immunization schedule [98–100]. The decline in the number of early-onset GBS SBIs has been ascribed to the screening of pregnant women in combination with intrapartum prophylactic antibiotic treatment recommended by several health authorities [98–100]. However, as reported by a Cochrane review [101], if effective, these measures reduce the incidences of early-onset GBS infection, whereas the epidemiology of late-onset GBS infection is not influenced. Regarding *Listeria monocytogenes* infection, antibiotic prophylaxis against GBS could have played a role in reducing the number of SBIs caused by this pathogen [102]. Penicillin G and ampicillin are the drugs of choice for prophylaxis against GBS [103], and for years, most *Listeria monocytogenes* strains have been found to be sensitive to ampicillin, although no evidence for ampicillin functioning as prophylaxis against this pathogen in pregnant women exists. However, the therapeutic association with gentamicin is suggested to assure effective intracellular concentrations [104]. However, available data have indicated a relationship between the decrease of early-onset GBS infection and the reduction of *Listeria monocytogenes* [105, 106] infection rates, although evidence of an impact on late-onset SBIs is lacking.

However, independent from the shifting reasons, the most important point regarding GBS antibiotic treatment is that emerging Gram-negative rods were recently found to be frequently resistant to ampicillin, particularly when cultured from children born to mothers who received this antibiotic for prophylaxis. This has raised concerns not only for the GBS prophylaxis antibiotic of choice but also for the treatment of children at high-risk for SBIs. In a relevant number of cases, use of the association between ampicillin and gentamicin, traditionally considered the drugs of choice to treat bacterial infections in neonates and younger infants, leaves a child with only gentamicin treatment [12, 20]. Because the use of the third generation cephalosporins is debatable, at least in neonatology, due to possibly increased neonatal mortality when these drugs are used as empirical therapy [107], only a continuous and detailed analysis of the local resistance patterns of common infectious pathogens can lead to effective antibiotic prescriptions.

## 6. Conclusions

The selection of neonates and young infants who are <3 months old with FWS who are at risk for SBI remains a problem without a definitive solution. The old Rochester criteria and the protocols derived from this remain effective for identifying young infants between 29 and 60 days old who do not have SBIs. The risk that an invasive disease is misdiagnosed is very low, especially today when the incidences of bacteremia and meningitis are reduced and most SBIs are UTIs. However, a more complex approach including the use of CRP and PCT can permit to identify a greater number of children with FWS that really have SBI and need immediate prompt hospitalization and adequate therapy. The step-by-step protocol previously cited seems presently the best solution to assure an adequate approach to all the children < 3 months with FWS. However, it is highly likely that, in the future, more effective methods to differentiate low- and high-risk children with FWS such as molecular techniques and new effective biomarkers will become available.

The approach in evaluating neonates is significantly more complicated, as their risk of SBIs, including bacteremia and meningitis, remains relevant and none of the suggested approaches can reduce the risk of dramatic mistakes. This is the main reason that most experts suggest obtaining cultures and prescribing immediate antibiotic treatment for neonatal children with FWS. However, the best antibiotic must be carefully selected considering the changing epidemiology and increasing antibiotic resistance of the most common infectious bacteria.

## Figures and Tables

**Table 1:** Historical criteria used in young infants with fever without a source (FWS) to identify those at risk of severe bacterial infection (SBI).

Approach	Clinical and laboratory criteria	Limits
Rochester criteria (‘80s)	Low risk of SBI in infants who appeared well (i.e., absence of tachypnea, dyspnea, tachycardia, bradycardia, lethargy, and decreased activity/appetite), had no evidence of ear, soft tissue, or skeletal infections, and had WBC counts between 5000 and 15,000/mm^3^, bands less than 1500/mm^3^, and ≤10 WBC per HPF. Moreover, in cases with diarrhoea, SBI could be excluded if ≤5 WBC/HPF could be observed in the stool	A relevant number of children without clinical problems considered at risk of SBI; not applicable in premature infants and in those with underlying medical condition
Philadelphia, Boston, and Milwaukee criteria (‘90s)	Clinical criteria, blood tests, and cut-off levels similar to those indicated in the Rochester protocol plus CSF testing and chest radiograph for the identification of patients at risk of SBI	Results similar to those observed with Rochester criteria, although management appeared more complicated with these protocols
Baraff criteria (‘90s)	Inclusion of a complete evaluation for sepsis with blood, urine, and CSF culture in neonates	Limited advantages with the use of universal CSF testing

CSF: cerebrospinal fluid; HPF: high-power field; SBI: severe bacterial infection; WBC: white blood cell.
